# Case Report: An early-onset inflammatory colitis due to a variant in *TNFAIP3* causing A20 haploinsufficiency

**DOI:** 10.3389/fped.2022.1044007

**Published:** 2022-11-18

**Authors:** Laura Zanatta, Francesca Biscaro, Silvia Bresolin, Maurizio Marzaro, Samantha Sarcognato, Ivana Cataldo, Antonio Marzollo, Stefano Martelossi

**Affiliations:** ^1^ Department of Women’s and Children’s Health, University Hospital of Padua, Padua, Italy; ^2^ Pediatric Department, Ca’ Foncello Hospital, Treviso, Italy; ^3^ Pediatric Surgery Department, Ca’ Foncello Hospital, Treviso, Italy; ^4^ Pathological Anatomy and Cytopathology Department, Ca’ Foncello Hospital, Treviso, Italy; ^5^ Pediatric Hematology, Oncology and Stem Cell Transplant Division, Padua University Hospital, Padua, Italy

**Keywords:** colitis, haploinsuffciency, HA20, anemia, TNFAIP 3, autoinflammatory diseases (AID), autoimmune diseases (AD)

## Abstract

Autoinflammatory diseases (AID) are a heterogeneous group of inherited conditions caused by abnormal activation of systems mediating innate immunity. Recent literature focuses on A20 Haploinsufficiency, an autoinflammatory disease with a phenotype resembling Behçet disease (BD). It is caused by *loss-of-function* mutations in *TNFAIP3* gene that result in the activation of a pro-inflammatory pathway. In this case report we describe a one-year-old baby who came to our attention for hematochezia appeared at three months of age which was considered an expression of early-onset colitis. The following appearance of cutaneous inflammation Behçet-like and the positive family history concurred with the diagnosis of an autoinflammatory disease. Extended genetic tests in the patient allowed to identify a heterozygous variant in *TNFAIP3* [NM_006290.4:c.460G > T, p.(Glu154Ter)], not previously described and not present in the GnomAD database. As a consequence the diagnosis A20 Haploinsufficiency was established and the appropriate management was started. The same *TNFAIP3* variant was also found in her father who had suffered from recurrent oral aphthosis, vitiligo and thyroiditis since childhood. In conclusion, we described a young patient with a novel heterozygous mutation in *TNFAIP3* who developed BD-like symptoms. We proposed that loss-of-function variants in *TNFAIP3* may be associated with a very early-onset intestinal BD phenotype.

## Introduction

Autoinflammatory diseases (AID) are a heterogeneous group of inherited conditions caused by abnormal activation of systems mediating innate immunity (1).

A20 Haploinsufficiency (HA20) is a recently described autoinflammatory disease with a phenotype resembling Behçet disease (1, 2). It is caused by loss-of-function mutations in the TNF *Alpha Induced Protein 3 (TNFAIP3)* gene encoding A20 that result in the activation of the pro-inflammatory pathway of nuclear factor (NF)-kB (3, 4).

Patients may present with dominantly inherited, early-onset systemic inflammation and a Behçet-like disease or a variety of autoinflammatory and autoimmune features (1, 2, 5).

In this case report, we describe a case of non-specific colitis as the first manifestation of A20 Haploinsufficiency.

## Case report

A one-year-old caucasian baby girl came to our attention for severe normocytic anemia, without signs of hemodynamic failure. From 3 months of age, our patient had a history of hematochezia during exclusive maternal breastfeeding. This condition was diagnosed as allergic proctitis and a maternal diet free of milk and derivatives was started. The weaning was conducted in a dairy-free diet but a complete resolution never occurred (6, 7).

**Figure 1:**
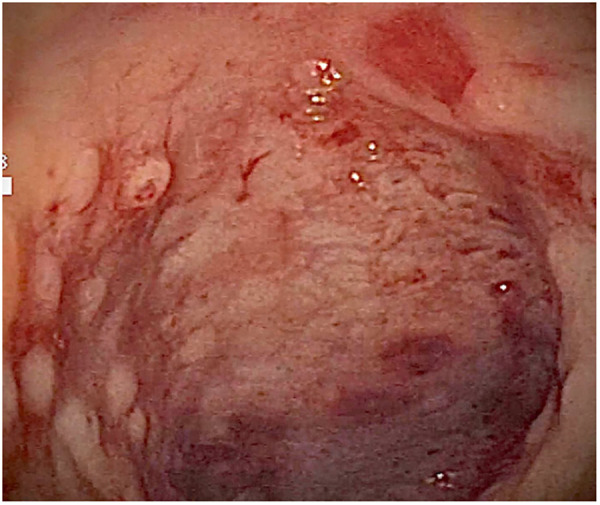
Ulcerated and bleeding colonic mucosa with petechial hemorrhages.

**Figure 2:**
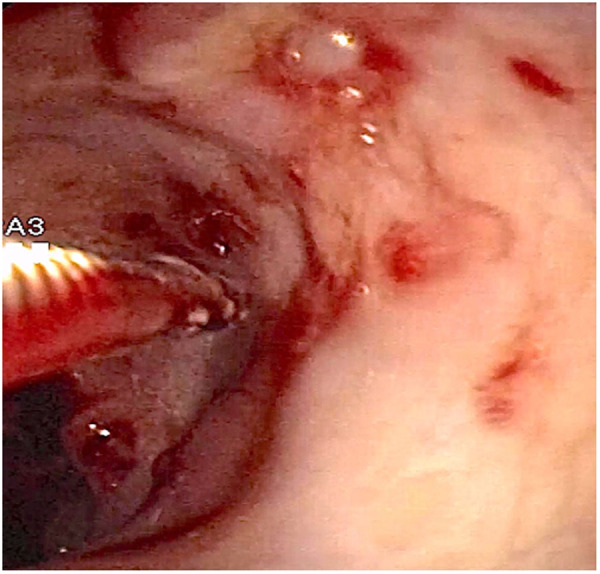
Fragile colonic mucosa with petechial hemorrhages.

**Figure 3:**
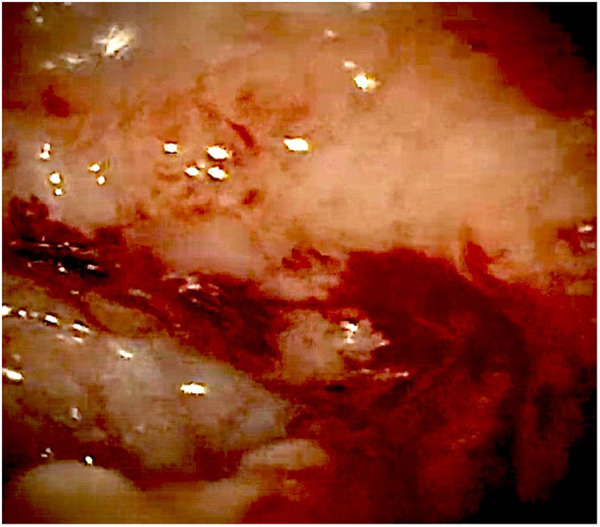
Bleeding colonic mucosa.

At admission to our Center, the first hypothesis was anemia due to gastrointestinal bleeding so the baby performed a colonoscopy, which revealed a macroscopic picture suggestive of colonic lymphoid nodular hyperplasia with ulcerated and bleeding mucosa (Figures 1–3).

**Figure 4:**
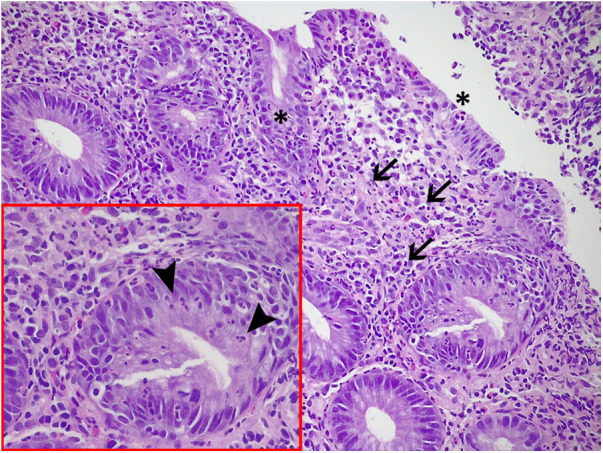
Histological examination of colonic biopsies: active colitis, with mucosal microerosions. (asterisks), a moderate inflammatory infiltrate in the lamina propria, including lymphocytes, plasmacells, and neutrophils (arrows), and an associated multifocal cryptitis, defined by the presence of intraepithelial neutrophils in colonic crypts (arrowheads in the box).

The histological examination revealed the presence of active colitis with no signs of chronicity and so the diagnosis of allergic colitis was excluded (Figure 4).

Since the genetic definition was not available, during the initial hospitalization we started only intravenous steroid therapy (methylprednisolone 2 mg/kg daily). At discharge, we continued an oral steroid therapy (betamethasone 0.2 mg/kg daily) without other immunosuppressive agents.

Moreover, in consideration of the low values of hemoglobin, she started an oral iron supplementation therapy and a gastroenterological follow-up was scheduled.

However, two weeks later, during steroid withdrawal, she came to the Emergency Room for acute panniculitis of the limbs without red flags (normal vital signs, good general condition with normal cardiovascular, thoracic and abdominal physical examination) and an anti-inflammatory therapy was prescribed.

**Figure 5:**
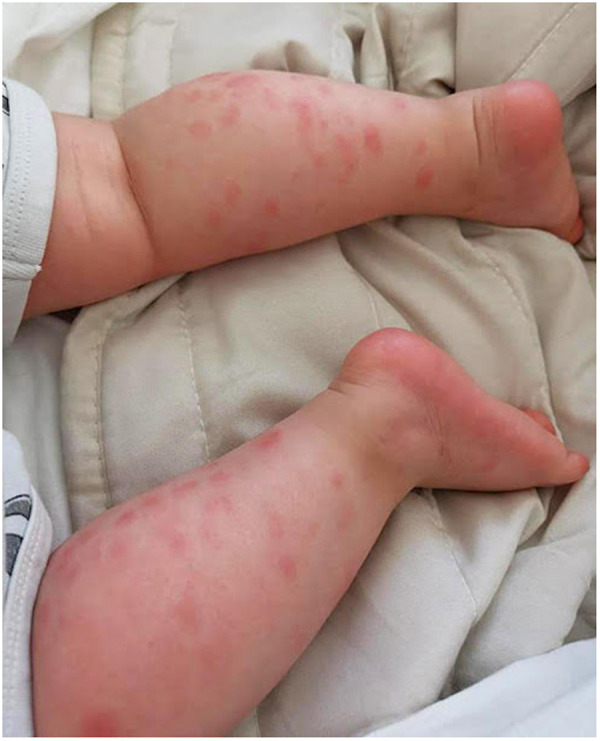
Panniculitis of the lower limbs.

**Figure 6:**
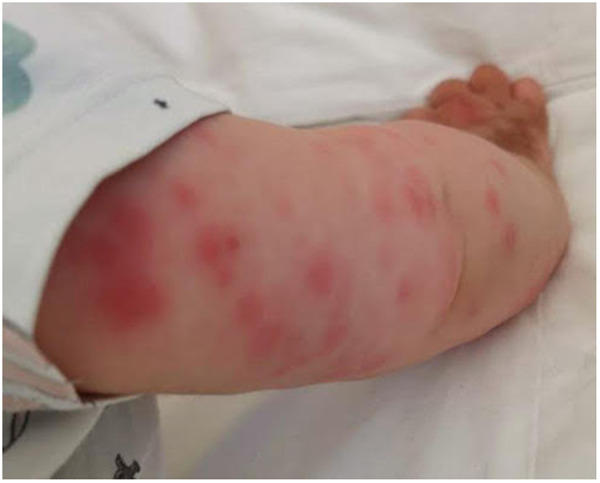
Panniculitis of the upper limbs.

**Figure 7:**
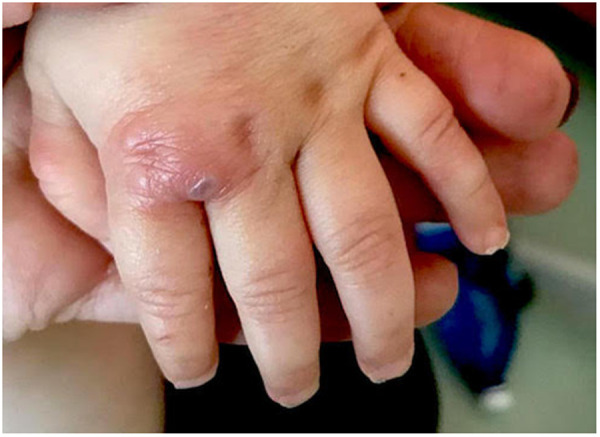
Ulcerative lesion of the hand.

**Figure 8:**
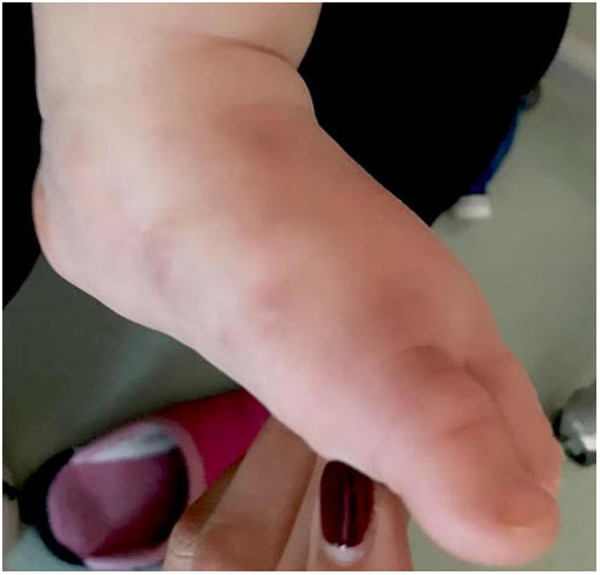
Necrotic lesion of the foot.

After only 48 h the skin condition worsened and the patient became febrile and suffering. On physical examination baby girl presented ulcerative and necrotic lesions associated with painful edema of the limbs (Figures 5–8).

Therefore, she was hospitalized for further investigations and intravenous steroid therapy was started. Blood tests showed a rise in inflammation indexes (WBC 35,000/µl with neutrophils prevalence and CRP 28 mg/dl, serum amyloid 100 mg/L) but the exams for infectious disease and neoplastic conditions were all negative. First-level autoimmune tests (VES, complement system, immunoglobulins, TNF, lymphocyte subpopulations, ASCA and ANCA antibodies) were all normal. The biopsy of the skin lesions showed leukocyte exocytosis of the epidermis and histiocytic and lymphocytic inflammation of the dermis. The eye examination was negative, in particular for uveitis. Fecal occult blood test (FOBT) turned out positive in three different samples and fecal calprotectin was elevated (>1,000 µg/g). IFN signature was normal and ADA-2 deficiency was excluded. Considering the laboratory exams and the early-onset gastrointestinal manifestations, the possibility of very early-onset inflammatory bowel disease (VEO-IBD) was evaluated so blood tests were performed but genetic studies were reported negative (8).

The patients had a family history of autoimmune conditions that involved the paternal line: her father with recurrent oral aphthosis, vitiligo and thyroiditis, her father's brother with recurrent oral aphthosis and vitiligo, a paternal grandfather with liver disease started when he was young, brother of paternal grandfather died at 16 years old due to unknown disease and father's uncle with Crohn's disease.

**Figure 9:**
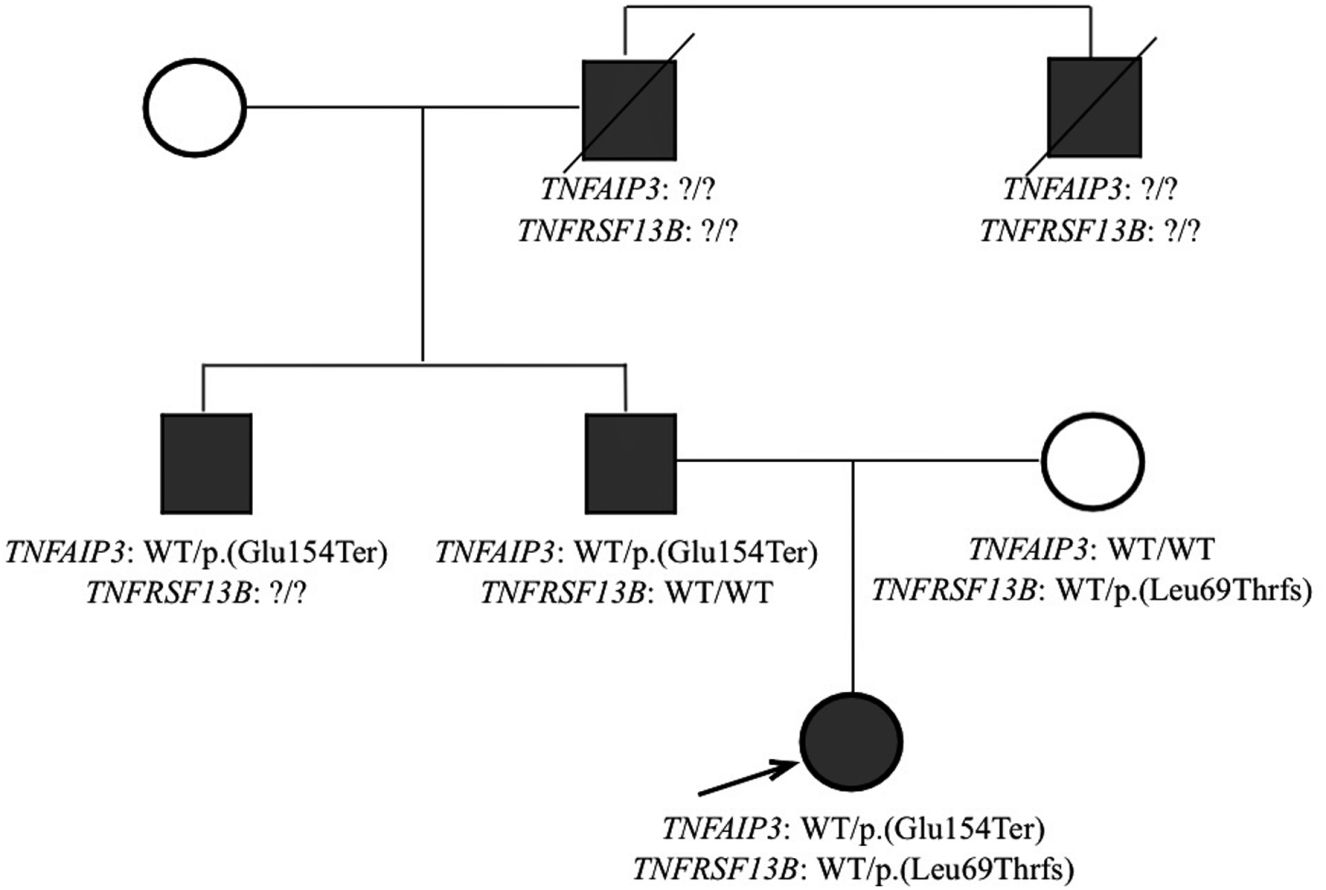
Family tree.

A custom gene panel sequencing including all genes included in the IUIS classification (9, 10) was employed to explore the genetic causes of the clinical phenotype, as previously described (11–13). We identified a heterozygous variant in *TNFAIP3*, namely NM_006290.4:c.460G > T, resulting in a premature stop codon *p*.(Glu154Ter). This variant was not previously described and is not present in the GnomAD database. Based on the characteristics of the variant, the genetic diagnosis of A20 haploinsufficiency was established. Additionally, a heterozygous known risk allele in *TNFRSF13B,* NM_012452.3:c.204dupA, p.(Leu69ThrfsTer12), was identified (14). Family segregation studies demonstrated the paternal origin of the *TNFAIP3* variant and the maternal origin of the *TNFRSF13B* allele. Family members truly screened for mutations in TNFAIP3 were the baby girl's mother and father. Later, the father's brother performed genetic test and identified the same mutations in TNFAIP3. The other family members in the paternal line presented potential clinical conditions of HA20 but could not be screened for mutations (Figure 9).

Initially, our patient was treated with a combination protocol, including systemic corticosteroids (betamethasone 0.1 mg/kg) and IL1 receptor antagonist (Anakinra from 2 to 6 mg/kg).

In consideration of the treatment-refractory disease with persistent gastrointestinal and skin manifestations, we shifted to an anti-TNF drug (Infliximab from 5 to 10 mg/kg, initially once a week and later every six weeks) in association with Methotrexate (7 mg once a week) (15).

Moreover, we decided to stop steroid therapy because of the important side effects (weight gain, changes in mood and behaviour, diabetes.).

After an initial resolution of clinical manifestations, two months later our patient presented a new relapse with unpainful skin lesions and widespread arthralgia. As a consequence, we decided to shift to Adalimumab (20 mg/kg every two weeks) in association with Methotrexate with good clinical response and rapid laboratory outcomes.

Skin and gastrointestinal manifestations resolved and blood tests known as inflammatory markers were negative, detecting a complete resolution of the inflammatory condition.

## Discussion

HA20 is an autoinflammatory disease due to a loss of function of A20 protein, whose role is to down-regulate the pro-inflammatory pathway of NF-kB (3, 4). It is inherited as an autosomal dominant trait, with early-onset and with very heterogeneous expressivity even within the same family (1, 2, 5). This condition is often called Behçet-like, with which it shares some features: recurrent painful oral and/or genital ulcers, musculoskeletal pains such as polyarthritis and/or arthralgia, gastrointestinal involvement with abdominal pain or mucosal bleeding, cutaneous lesions, episodic fever and recurrent infections (1, 5, 15). This variability also affects therapy: treatment regimens should be based on disease severity but it is difficult because of the lack of standardized protocol (15, 16).

In our case, the disease appeared at 3 months of age with indeterminate early onset colitis and later skin manifestations started. Probably the father, the paternal uncle and the other members of the family in the paternal line present the same condition, but with later and milder manifestations.

The variability of phenotypes in patients with HA20 is striking with a substantial difference also in the same family (16). In the family described here, the index patient presented with an infantile onset severe intestinal, joint and skin involvement, while the other affected family members presented later in life, with less severe clinical manifestations. We speculate that the coexistence of the maternally inherited risk allele in *TNFRSF13B* might have contributed to disrupting a potential compensatory mechanism in adaptive immunity. Indeed, digenic inheritance has been described in patients with proteasome-associated autoinflammatory syndromes (PRAAS) and relatively common genetic variants can act as disease modifiers (17).

In literature our patient's clinical presentation, characterized by colitis and cutaneous manifestations, was already mentioned. Nevertheless, such important symptoms and the very early onset are two rare aspects, not described in other works.

According to the literature, patients with A20 Haploinsufficiency require treatment but at the moment a standardized therapeutic approach does not exist and every patient needs an individual treatment regimen (16). Some patients receive immunosuppressive drugs, others anti-cytokine agents and patients with severe disease go into haematopoietic stem cell transplant.

Based on studies, because of the very early-age of onset, the primary treatment is anti-interleukin 1 (Anakinra) but the remission was partial. Then we attempted to use TNF-antagonist (Infliximab) and Methotrexate with a temporary response. Finally, we shifted to Adalimumab and Methotrexate with rapid and good outcomes (15, 16). As far as concern therapeutic strategy, an interesting work reported that a type I interferon (IFN) signature or elevation of IFN-stimulated genes (ISGs) predicts a positive response to JAK-inhibition. In our case, this could not be applied because the interferon signature of the patient was normal (18).

## Conclusion

In conclusion, we presented a HA20 patient with gastrointestinal BD-like symptoms and identified a novel heterozygous variant (c.460G > T, p.Glu154Ter) in *TNFAIP3*.

Based on our observation, HA20 should be considered in patients with infantile and very early-onset mucosal or cutaneous inflammation, especially with positive family history and genetic screening for *TNFAIP3* could be evaluated for patients with BD-like symptoms.

## Data Availability

The raw data supporting the conclusions of this article will be made available by the authors, without undue reservation.
